# Philadelphia College of Medicine

**Published:** 1854-11

**Authors:** 


The Philadelphia College of Medicine has been reorganized with an able and distinguished faculty. This is the school disgraced by McClintock, and which, prior to his apostacy, was not in especially good odor. It is now, however, in the hands of men capable of conducting a good school, and its former name should do it no injury. We wish it an honorable prosperity. H.

